# Construction of novel mRNA-miRNA-lncRNA regulatory networks associated with prognosis of ovarian cancer

**DOI:** 10.7150/jca.49557

**Published:** 2020-10-17

**Authors:** Lingling Gao, Xiao Li, Xin Nie, Qian Guo, Qing Liu, Yue Qi, Juanjuan Liu, Bei Lin

**Affiliations:** 1 Department of Obstetrics and Gynaecology, Shengjing Hospital of China Medical University, No.36 Sanhao Street, Heping District, Shenyang, 110004, Liaoning, China.; 2 Key Laboratory of Maternal-Fetal Medicine of Liaoning Province, Key Laboratory of Obstetrics and Gynecology of Higher Education of Liaoning Province, Liaoning, China.

**Keywords:** ovarian cancer, competing endogenous RNA, prognosis, bioinformatics analysis

## Abstract

**Background:** Ovarian cancer (OC) is the most lethal malignancy in the female reproductive system. Growing evidences demonstrates that competing endogenous RNA (ceRNA) network play crucial roles in the occurrence and progression of tumors. Therefore, we aimed to explore and identify novel mRNA-miRNA-lncRNA ceRNA networks associated with prognosis of OC.

**Methods:** The differentially expressed gene (DEGs) of four expression profiles datasets (GSE5438, GSE40595, GSE38666 and GSE26712) were collected from Gene Expression Omnibus (GEO) database and analyzed with NetworkAnalyst. Intersection of DEGs were further employed for Gene Ontology (GO) and Kyoto Encyclopedia of Gene and Genome (KEGG) pathway analysis. Protein-protein interaction (PPI) network and hub genes of DEGs were also identified. The expression levels and survival analysis of the hub genes in OC and their upstream miRNAs and lncRNAs were performed by various bioinformatics databases. More importantly, ceRNA networks were constructed based on mRNA-miRNA-lncRNA in OC.

**Results:** A total of 178 DEGs including 38 upregulated and 140 downregulated genes from intersected DEGs of four expression profiles were identified in OC. Functional enrichment analysis suggested that the commonly DEGs were enriched in regulating enzyme inhibitor activity, glycosaminoglycan and G protein-coupled receptor binding, cell morphogenesis, and involved in pathways including metabolic process, proteoglycans in cancer. Top 10 hub genes with higher connectivity degree were selected for subsequent expression and prognosis analysis. After take expression levels and prognostic roles of hub genes and their upstream miRNAs and lncRNAs in OC into consideration, 2 mRNAs (TACC3 and CXCR4), 2 miRNAs (hsa-miR-425-5p and hsa-miR-146a-5p) and 3 lncRNAs (FUT8-AS1, LINC00665 and LINC01535) were significantly associated with the poor prognosis of OC. The mRNA-miRNA-lncRNA networks (TACC3-hsa-miR-425-5p-FUT8-AS1 and CXCR4-hsa-miR-146a-5p-LINC00665/LINC01535) were eventually constructed in OC based on ceRNA mechanism.

**Conclusion:** We successfully constructed novel ceRNA network associated with the prognosis of ovarian cancer, which may provide a new strategy for early diagnosis and therapeutic intervention of OC.

## Introduction

Ovarian cancer is one of the most common malignant tumors in gynecology worldwide 1, which causes the highest mortality and poor prognosis due to the low diagnostic accuracy in the early stage and extensive metastasis at an advanced stage 2. Although the great advancements in therapeutic strategies of ovarian cancer has been achieved including surgery, chemotherapy and radiotherapy, the 5-year survival rate of ovarian cancer patients was still less than 45% 3, 4. Effective biomarkers for early diagnosis and prognosis evaluation have not been fully explored and clarified. Therefore, a better understanding of molecular mechanisms that associated with the prognosis of ovarian cancer may contribute to the development of advanced diagnostic and therapeutic technologies to improve the survival quality of patients with ovarian cancer.

Accumulating evidences have demonstrated that noncoding ribonucleic acids (ncRNA) play key roles in the occurrence and development of multiple tumors, including microRNAs (miRNAs), long noncoding RNA (lncRNAs) and circular RNA (circRNA) 5. Based on different transcripts in length with no or limited protein-coding ability, both miRNAs and lncRNAs could not only participate in a variety of biological processes and molecular mechanism of tumors, such as regulating gene transcription and post-transcriptional translation 6, epithelial-to-mesenchymal transition 7 and signaling pathways 8, but exert profound influence on early diagnosis, prognosis evaluation and therapeutic targets of different malignancies including ovarian cancer 9. Recent studies have suggested that lncRNAs, based on the competitive endogenous RNA (ceRNA) mechanism, can competitively bind to miRNAs acting as sponge of miRNAs to further relieve the suppression of miRNAs on their target genes 10. The aberrant regulation of mRNA-miRNA-lncRNA ceRNA network play key role in tumorigenesis and progression of multiple cancers, such as gastric cancer 11, breast cancer 12 and ovarian cancer 13. However, the underlying mechanisms of mRNA-miRNA-lncRNA ceRNA regulatory networks, especially associated with prognosis of ovarian cancer, are not fully clarified.

In this present study, we collected four original expression profiles by array (GSE5438, GSE40595, GSE38666 and GSE26712) of ovarian cancer from the Gene Expression Omnibus (GEO) database. Differentially expressed genes (DEGs) between ovarian cancer and normal samples were further identified with various bioinformatics approaches. Additionally, the intersection of DEGs of these four datasets were further employed for functional enrichment analysis, protein-protein interaction (PPI) network construction and top-ranked hub genes identification with online tools. After comprehensive evaluation of expression levels and prognostic roles of hub genes in ovarian cancer, 4 upregulated DEGs and 1 downregulated DEGs were eventually identified for predicting their upstream miRNAs with the miRTarBase online database. Furthermore, the upstream miRNAs which analyzed by expression levels and prognostic values were chosen for predicting the upstream lncRNAs by miRNet database. Similarly, the expression levels and prognostic values of these upstream lncRNAs were also analyzed with online database. As a consequence, an mRNA-miRNA-lncRNA regulatory network associated with the prognosis of patients with ovarian cancer was successfully constructed. This study may provide new insights into exploring and identifying novel diagnostic biomarkers or potential targets for therapeutic intervention of ovarian cancer.

## Materials and Methods

### Collection of datasets

Four expression profiles by array (GSE54388, GSE40595, GSE38666 and GSE26712) of ovarian cancer were collected from the Gene Expression Omnibus (GEO) (www.ncbi.nlm.nih.gov/geo/) online database 14. According to publication time and the sample size, only datasets being published the past 10 years and including at least 10 epithelial ovarian cancer samples were enrolled in current research. Datasets with only blood samples or cell lines of ovarian cancer were excluded, and patients with chemotherapy, radiotherapy, hormone therapy before surgery and lack of histopathological diagnosis were not implemented in this study. The datasets from GSE54388 15, GSE40595 16 and GSE38666 17 were all based on the GPL570 platform (HG-U133_Plus_2; Affymetrix Human Genome U133 Plus 2.0 Array). GSE54388 dataset covered 16 ovarian cancer samples and 6 normal ovarian epithelium samples. GSE40595 dataset contained 32 ovarian epithelial tumor samples and 6 normal ovarian epithelium samples. GSE38666 dataset contained 18 ovarian cancer patients and 12 ovarian surface epthelium samples. GSE26712 18, which was based on the GPL96 ([HG-U133A] Affymetrix Human Genome U133A Array), contained 185 primary ovarian tumors and 10 normal ovarian epithelium samples. We further downloaded the platform and series matrix files of above four datasets.

### Identification of differentially expressed genes (DEGs)

NetworkAnalyst 3.0 (https://www.networkanalyst.ca/) 19 is a user-friendly bioinformatics tools that helps to perform comprehensive gene expression analysis, meta-analysis and network analysis, which accepts five types of data inputs including one or multiple gene lists, a single or multiple gene expression data, raw RNAseq reads as well as series matrix files. This unique online tool integrates cell or tissue-specific protein-protein interactions (PPI), TF-gene interaction networks, miRNA-gene interactions, protein-drug interactions and protein-chemical interactions, and the processes of which contain data update, data processing and analysis, integrated knowledgebase and interactive visual analysis. DEGs can be identified with statistical methods such as limma, edgeR and DESeq2. In this study, NetworkAnalyst 3.0 was employed to normalize the data and identify DEGs in each dataset, the cut-off criteria were set as follows: adjusted *P* < 0.05 and log2 fold change (log2 FC) ≥1.

### Functional enrichment analysis

All the DEGs of the four datasets identified by NetworkAnalyst 3.0 were further divided into upregulated DEGs and downregulated DEGs, respectively. The commonly DEGs in all of the various datasets were subjected to subsequent analysis. An online tool-Draw Venn Diagram (http://bioinformatics.psb.ugent.be/webtools/Venn/) was employed to explore the intersection genes of the four datasets by venn diagrams. To further explore the potential functions and mechanisms of above commonly DEGs in ovarian cancer, Metascape (http://metascape.org) 20 was used to conduct Gene Ontology (GO) and Kyoto Encyclopedia of Genes and Genomes (KEGG) pathway enrichment analysis of all the commonly DEGs. The thresholds: *P* <0.05, a minimum count of 3, enrichment factor >1.5 were considered to be statistically significant.

### Construction and analysis of PPI network and identification of hub genes

To explore the hub genes correlated with ovarian cancer, PPI networks of commonly DEGs identified (upregulated and downregulated DEGs) were constructed separately with the Search Tool for the Retrieval of Interacting Genes (STRING) (http://string-db.org) 21, which is a flexible and user-friendly platform that facilitates protein-protein interaction networks. A confidence score ≥ 0.4 was set as the cut-off criteria to construct PPI network. Subsequently, the hub genes of the PPI network were identified by the CytoHubba, a plug in Cytoscape software (v3.7.1) 22. Based on the degree of connectivity of DEGs, and the top 20 hub genes were exhibited separately with Cytoscape, and the top 10 hub genes were identified separately as hub genes for further analysis and validation.

### Validation of gene expression and prognostic values analyzed with GEPIA, HPA and Kaplan-Meier plotter

The Gene Expression Profiling Interactive Analysis (GEPIA) (http://gepia.cancer-pku.cn/) 23 is an effective web interface that covers gene expression data from 9736 tumor samples and 8587 normal samples. The web-based tool provides various analysis modules such as analyzing differential gene expression, evaluating survival and prognosis and correlation analysis. In this research, GEPIA database was utilized to further explore the expression of the top 10 hub genes identified in DEGs. One-way ANOVA was used to evaluate the differences of hub genes between tumor samples and normal samples, and the filter criteria were set as follows: *P*-value < 0.05, |Log2FC| >2. Human Protein Atlas (HPA) (https://www.proteinatlas.org/) 24 could provide the distribution, expression and prognosis of 24000 human proteins in 20 tumors tissues, 48 normal tissues, 47 cell lines and 12 blood cells validated by immunology method. In this study, HPA database was used to investigate the staining of hub proteins in ovarian cancer and normal tissues with immunohistochemistry. The Kaplan-Meier (KM) Plotter (http://kmplot.com) 25 is an online tool for evaluating the prognosis of patients with tumors including 2190 ovarian cancer samples. The hazard ratio (HR) at a 95% confidence interval and log-rank* P*-values were also explored online. The filter conditions were as follows: cancer: ovarian cancer; survival: progression free survival (PFS); follow-up threshold: 120 months, log-rank *P* value < 0.05 was regarded as statistically significant difference.

### Identification of upstream miRNA

miRTarBase (http://mirtarbase.mbc.nctu.edu.tw/php/index.php) 26, a newly web-based database, mainly contains miRNA-target interactions verified by different experiments and provides powerful evidences with literatures or assays. In this study, the upstream miRNAs of the hub genes were investigated by miRTarBase, and only those verified by at least one powerful experiment were identified as the potential miRNAs interactions (reporter assay, Western blot or quantitative reverse transcription PCR) and then chosen for subsequent analysis. dbDEMC (database of Differentially Expressed MiRNAs in human Cancers) (https://www.picb.ac.cn/dbDEMC/) 27 is an integrated database that designed to explore differentially expressed microRNAs (miRNAs) in human cancers detected by high-throughput methods, including a total of 209 newly published data sets collected from Gene Expression Omnibus (GEO) and The Cancer Genome Atlas (TCGA). We utilized this database to explore the expression of upstream miRNAs in ovarian cancer, and the prognostic values of miRNAs were detected by the Kaplan-Meier Plotter.

### Identification of upstream lncRNA

miRNet (https://www.mirnet.ca/) 28 is a user-friendly and online tool which provides miRNA-centric multiplex networks integrating key molecules of interest, and contains comprehensive interaction between miRNA and its targeted lncRNA. In this study, miRNet was employed to detect the potential upstream lncRNAs correlated with key miRNA, and the selection criteria were set as follows: Organism: Homo sapies, Target type: lncRNAs. What's more, the expression levels and prognostic values of these potential lncRNAs were further evaluated by GEPIA database and Kaplan-Meier Plotter. The lncRNAs conformed to the ceRNA hypothesis were identified as key lncRNA.

### Construction of the mRNA-miRNA- lncRNA regulatory network

LncLocator (https://LncLocatorwww.csbio.sjtu.edu.cn/bioinf/lncLocator/) 29 is a reliable online platform to analyze the subcellular localization of lncRNAs, which includes 5 subcellular localizations of lncRNAs and their distribution proportion, such as cytoplasm, nucleus, ribosome, cytosol and exosome. LNCipedia databases (https://lncipedia.org) 30 is a freely and effectively annotated database of lncRNAs transcriptional sequences and structures, which provides insights into functions of over 1500 human lncRNAs, including evaluating coding ability, predicting open reading frame and secondary structure. In this study, sequences information of lncRNAs were explored by LNCipedia databases, and the cellular localizations of lncRNAs were then detected by LncLocator. Cytoscape is a very powerful and effective software for visualizing and analyzing network data, which assists users to achieve many complex biological networks 22. Node and edge are the two core elements in the network diagram constructed by Cytoscape. Cytoscape was further employed to construct and visualize competing endogenous RNA (ceRNA) network (lncRNA-miRNA-mRNA), including differentially expressed genes, differentially expressed miRNAs, and differentially expressed lncRNAs.

## Results

### Identification of significant DEGs in ovarian cancer from GEO database

**Figure 1:**
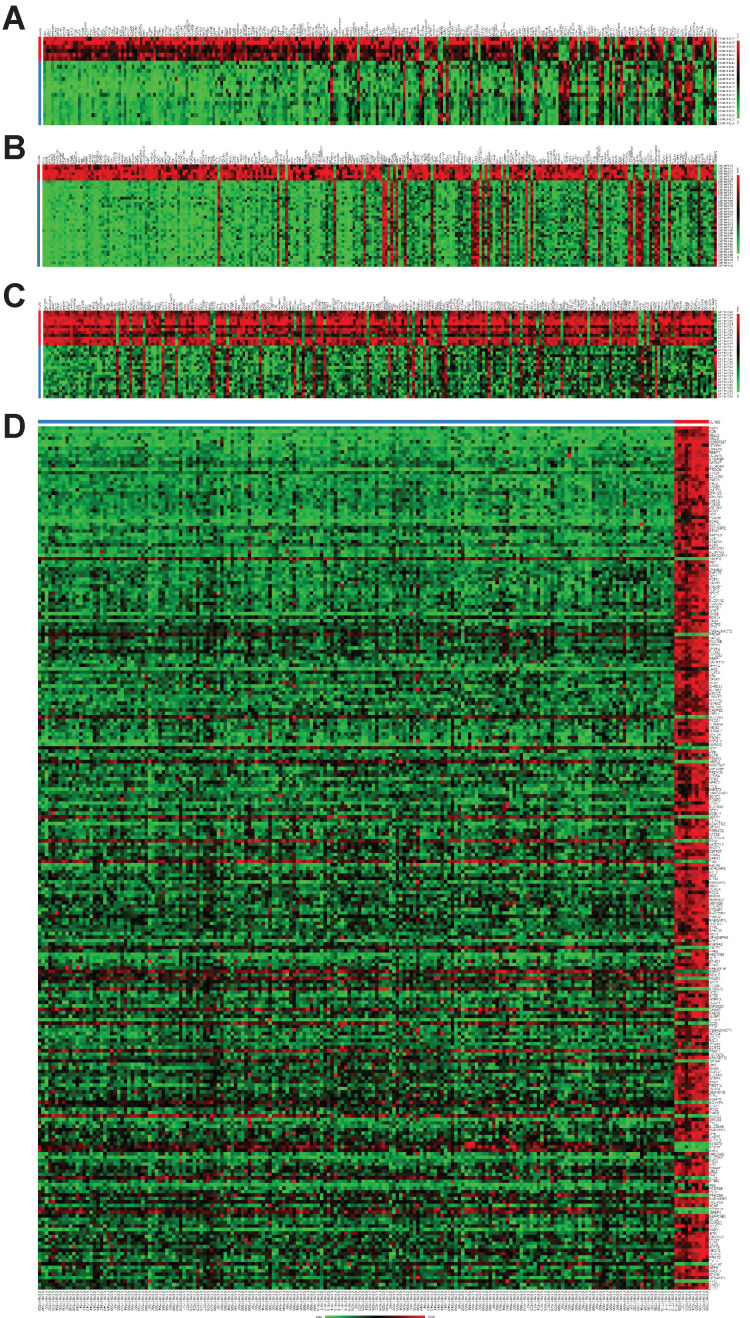

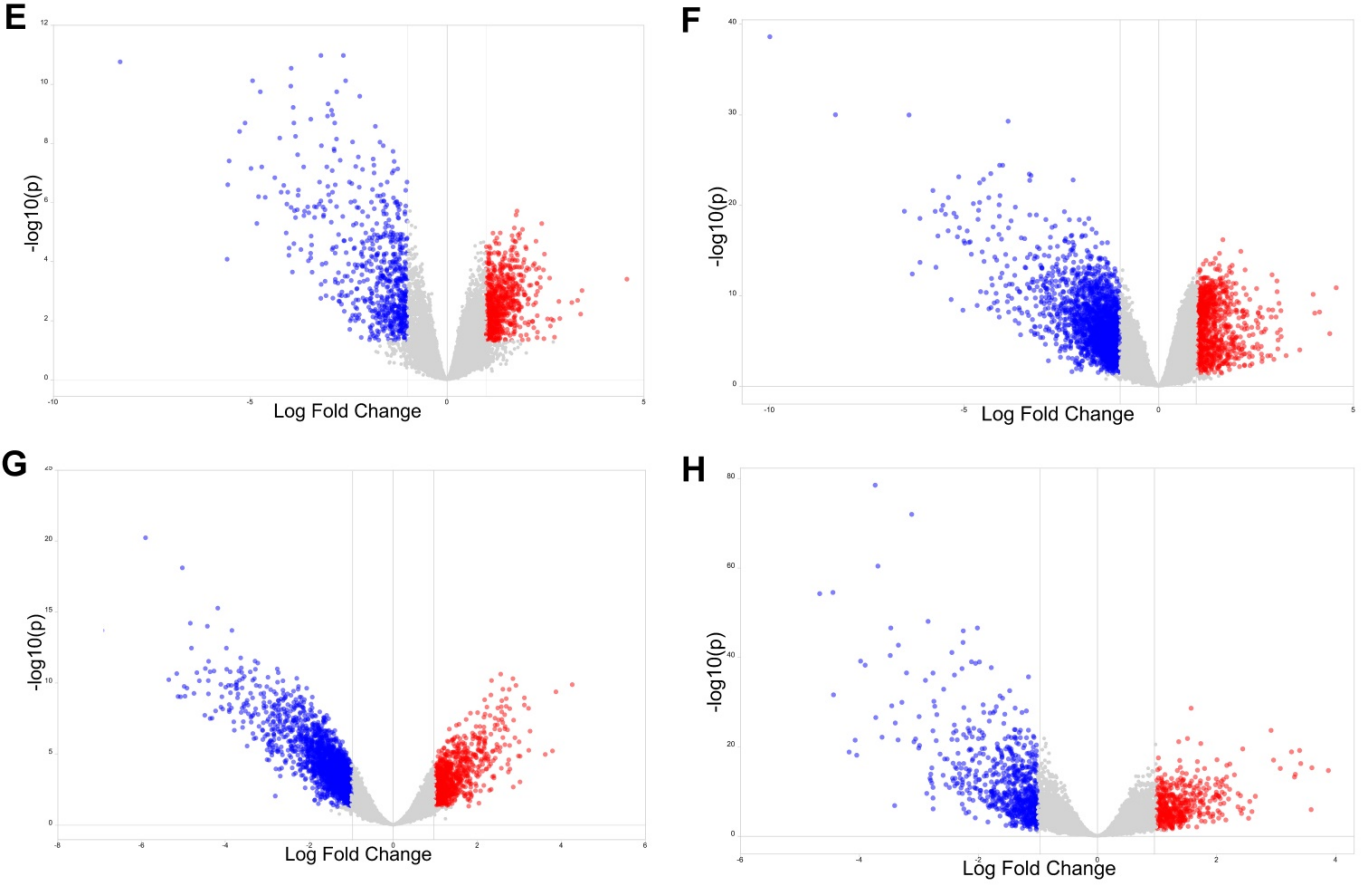
** Identification of differentially expressed genes (DEGs) in ovarian cancer from GEO datasets.** (A-D) The heatmap of DEGs (Top 250) in GSE54388 (A), GSE40595 (B), GSE38666 (C) and GSE26712 (D) datasets shown by NetworkAnalyst. Red: the upregulated genes; green: the downregulated genes. (E-H) The volcano plot of DEGs in GSE54388 (E), GSE40595 (F), GSE38666 (G) and GSE26712 (H) datasets shown by NetworkAnalyst. Red spots: the upregulated genes; blue plots: the downregulated genes. DEGs, differentially expressed genes.

To explore the potential roles of molecular associated with the tumorigenesis, development and prognosis of ovarian cancer, we firstly identified DEGs in four expression profiles (Table 1) downloaded from GEO with NetworkAnalyst. According to the pre-defined cut-off criteria (adjusted *P*<0.05 and |log2 FC| >1), as shown in the heatmaps and volcano plots, a total of 1420 DEGs (756 upregulated genes and 664 downregulated genes) were identified between ovarian cancer samples and normal samples from GSE54388 dataset (Figure 1A and Figure 1E). In GSE40595 dataset, a whole of 3101 DEGs (936 upregulated genes and 2165 downregulated genes) were screened out in ovarian cancer samples compared with normal samples (Figure 1B and Figure 1F). In GSE38666 dataset, a total of 3243 DEGs (1018 upregulated genes and 2225 downregulated genes) were identified in ovarian cancer samples compared with normal samples (Figure 1C and Figure 1G). In GSE26712 dataset, a total of 1200 DEGs (487 upregulated genes and 713 downregulated genes) were detected in ovarian cancer tissues compared with normal samples (Figure 1D and Figure 1H). The clinical characteristics of all patients with ovarian cancer in GSE38666 and GSE26712 were displayed in Supplementary Table S1 and Table S2, and detailed clinic parameters of enrolled patients in GSE54388 and GSE40595 were not provided in the original researches.

### Identification of DEGs in ovarian cancer shared by four GEO datasets and functional analysis for DEGs

**Figure 2:**
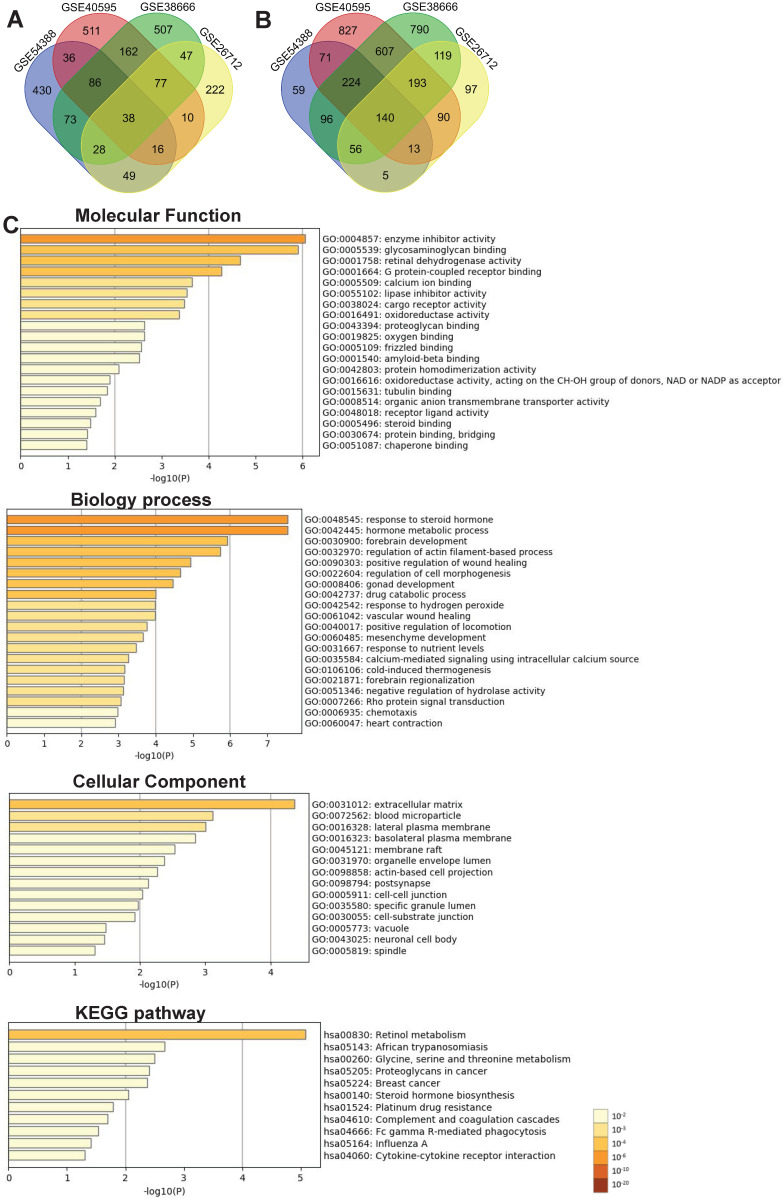
** Identification of DEGs between ovarian cancer and normal samples shared by four GEO datasets and functional analysis of the intersected DEGs with Metascape.** (A-B) The intersection of upregulated (A) and downregulated (B) DEGs in four GEO expression profiles with venn diagrams. (C) Significant enrichment analysis of GO and KEGG pathways of intersected DEGs colored by *P*-value with bar graph with Metascape. DEG: Differentially expressed gene; KEGG: Kyoto Encyclopedia of Genes and Genome.

We further performed the overlapping analysis of the upregulated or downregulated DEGs in four datasets separately with a total of 178 DEGs, including 38 upregulated (Figure 2A) and 140 downregulated genes (Figure 2B) were considered as commonly dysregulated genes in four GEO expression profiles shown by the Venn diagram. The result of detailed DEGs were shown in Supplementary Table S3 and used to further analysis. To investigate the potential functions and mechanisms of identified intersected DEGs in the development of ovarian cancer, GO and KEGG pathway enrichment analysis of intersected DEGs (including 38 up-regulated and 140 down-regulated genes) were explored by Metascape. The results of GO enrichment analysis showed that the intersected DEGs were mainly enriched in regulating enzyme inhibitor activity, retinal dehydrogenase activity, glycosaminoglycan binding and G protein-coupled receptor binding (Figure 2C and Supplementary Table S4). The biological processes of intersected DEGs were involved in response to steroid hormone, hormone metabolic process, regulation of actin filament-based process and cell morphogenesis (Figure 2C and Supplementary Table S5). The intersected DEGs were mainly focused on extracellular matrix, blood microparticle, lateral plasma membrane, basolateral plasma membrane (Figure 2C and Supplementary Table S6). KEGG enrichment analysis revealed that these intersected DEGs could participate in signaling pathways such as retinol metabolism, proteoglycans in cancer, glycine, serine and threonine metabolism (Figure 2C and Supplementary Table S7).

### PPI network construction and identification of hub genes

**Figure 3:**
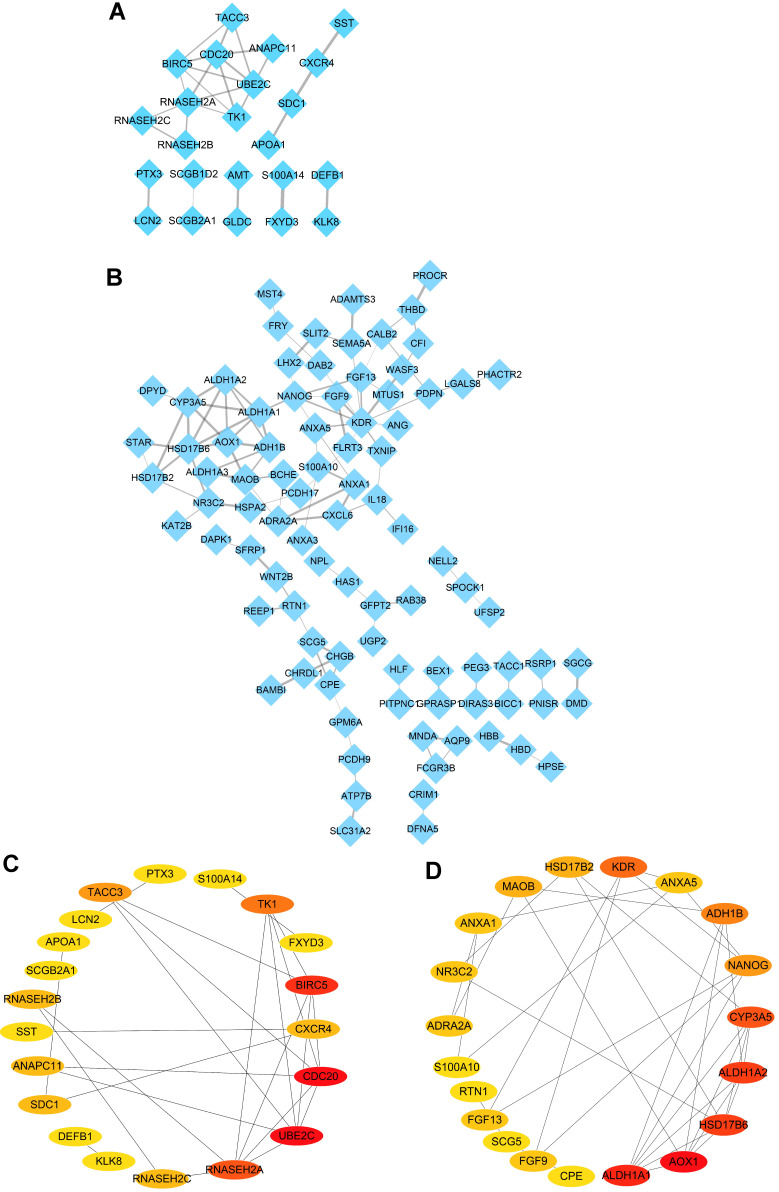
** Identification of hub genes in ovarian cancer with Cytoscape software.** (A-B) PPI networks of upregulated (A) and downregulated (B) DEGs constructed by Cytoscape software, respectively. (C-D) The significantly upregulated genes (C) and downregulated genes (D) (top 20 hub genes) shown by PPI network, respectively.

In order to further explore the protein-protein interaction of the identified DEGs, the STRING online tool was employed to investigate the relationship among the 38 upregulated DEGs and 140 downregulated DEGs in the intersection of four datasets, and all the data were further extracted and visualized by PPI networks constructed with Cytoscape software. The result showed complicated interactions among these intersected genes (Figure 3A-B). Subsequently, the top 20 upregulated and downregulated hub genes shared by four datasets were screened out and visualized with CytoHubba plug in Cytoscape software (Figure 3C-D). What's more, the top ten upregulated DEGs (UBE2C, CDC20, BIRC5, RNASEH2A, TK1, TACC3, CXCR4, SDC1, RNASEH2B and RNASEH2C) (Table 2) and top ten downregulated DEGs (KDR, HSD17B6, NANOG, AOX1, CYP3A5, ALDH1A1, ADH1B, MAOB, ALDH1A2 and FGF13) (Table 2) were regarded as hub genes in ovarian cancer and selected for the following investigation.

### Validation of gene expression of hub genes and survival analysis

**Figure 4:**
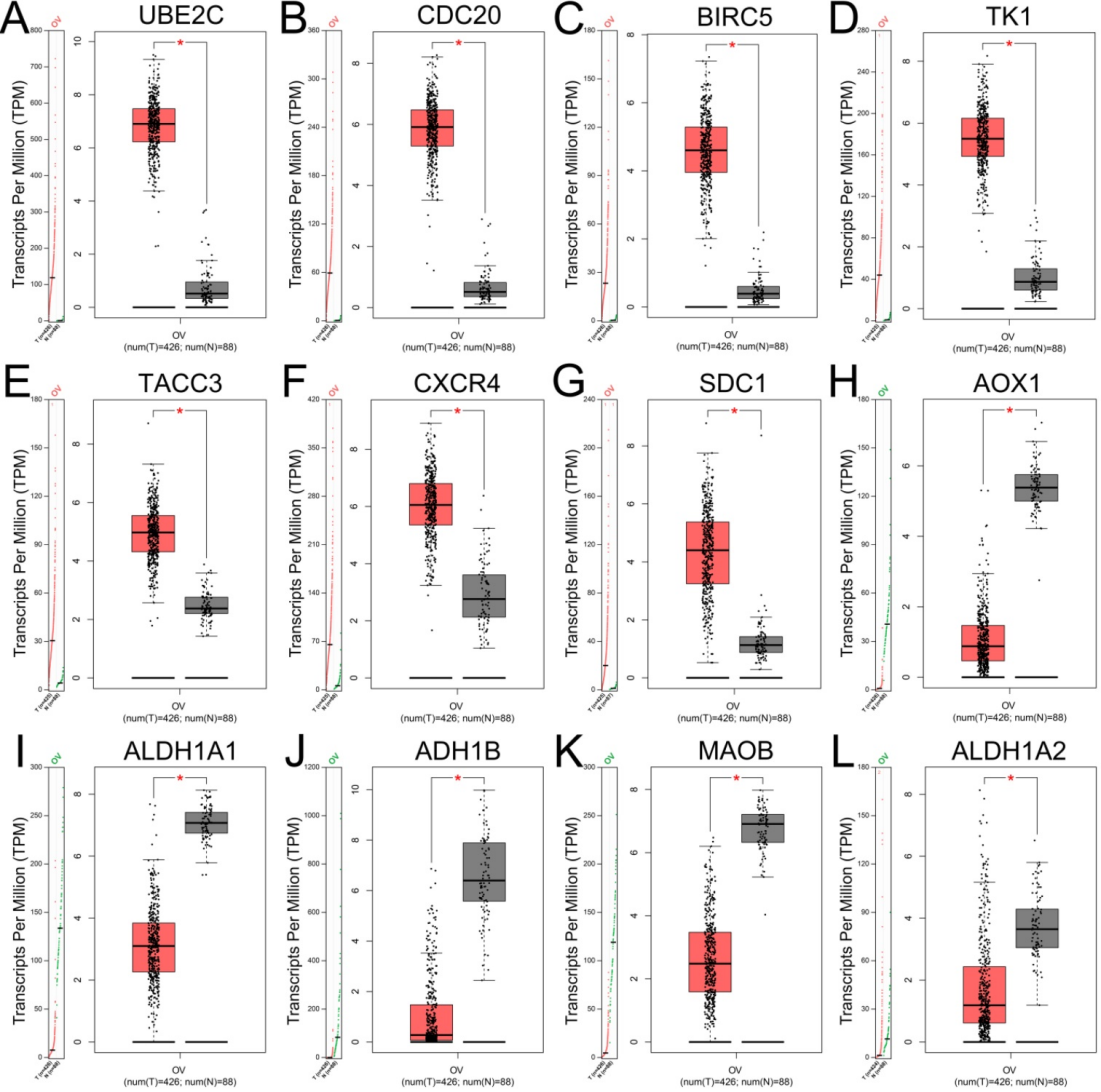
** Expression levels of hub genes in patients with ovarian cancer validated with GEPIA.** (A-G) The expression levels of upregulated hub genes UBE2C (A), CDC20 (B), BIRC5 (C), TK1 (D), TACC3 (E), CXCR4 (F) and SDC1 (G) in ovarian cancer compared with normal tissues (all *P*<0.05). (H-L) The expression levels of downregulated hub genes AOX1 (H), ALDH1A1 (I), ADH1B (J), MAOB (K) and ALDH1A2 (L) in ovarian cancer compared with normal tissues by GEPIA (all *P*<0.05). TPM: Transcripts per Million.

**Figure 5:**
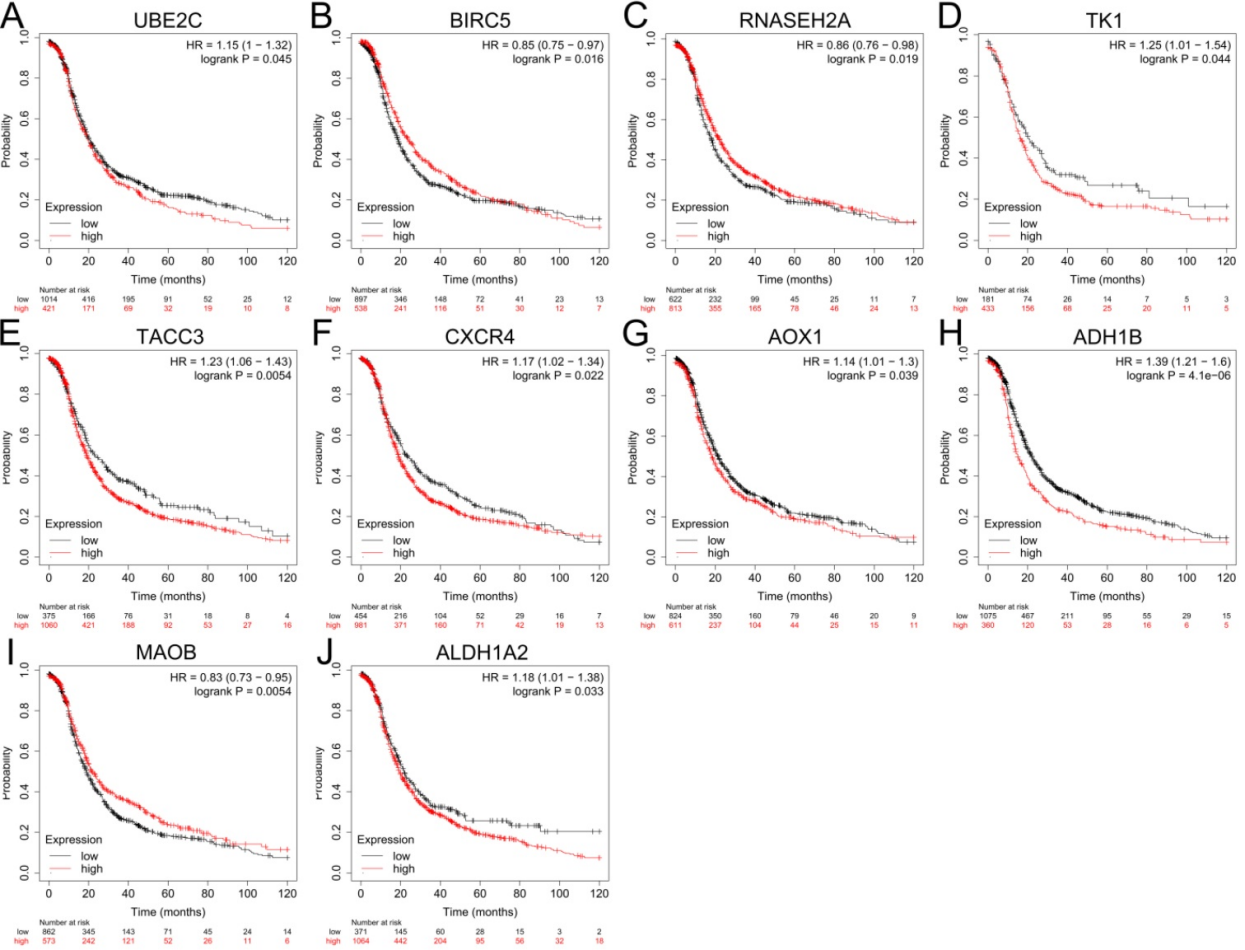
** Prognostic values of hub genes in ovarian cancer analyzed by using the Kaplan-Meier plotter.** (A-F) Relationship between UBE2C (A), BIRC5 (B), RNASEH2A (C), TK1 (D), TACC3 (E), CXCR4 (F) and PFS of patients with ovarian cancer. (G-J) Relationship between AOX1 (G), ADH1B (H), MAOB (I), ALDH1A2 (J) and PFS of patients with ovarian cancer by Kaplan-Meier plotter. PFS: progression free survival.

The expression levels of top 10 upregulated and downregulated hub genes were validated by the GEPIA database (Figure 4) and the survival analysis of those hub genes for progression free survival (PFS) in patients with ovarian cancer were explored by online tool Kaplan-Meier plotter (Figure 5). the result showed that UBE2C (Figure 4A), CDC20 (Figure 4B), BIRC5 (Figure 4C), TK1 (Figure 4D), TACC3 (Figure 4E), CXCR4 (Figure 4F) and SDC1 (Figure 4G) were upregulated in ovarian cancer compared with normal group (all *P*<0.05), and there were no significant differences in the expression of RNASEH2A, RNASEH2B and RNASEH2C between ovarian cancer samples and normal samples (Supplementary Figure S1A-C). For the downregulated hub genes, the expression of AOX1 (Figure 4H), ALDH1A1 (Figure 4I), ADH1B (Figure 4J), MAOB (Figure 4K) and ALDH1A2 (Figure 4L) were significantly suppressed in ovarian cancer compared with normal samples, and there were no significant differences in the expression of KDR, HSD17B6, MANOG, CYP3A5 and FGF13 between ovarian cancer samples and normal samples (Supplementary Figure S1D-H). After validation of gene expression and evaluation of prognostic values of hub genes, only high expression of UBE2C (*P*=0.045, HR=1.15 [1-1.32], Figure 5A), TK1 (*P*=0.044, HR=1.25 [1.01-1.54], Figure 5D), TACC3 (*P*=0.0054, HR=1.23 [1.06-1.43], Figure 5E) and CXCR4 (*P*=0.022, HR=1.17 [1.02-1.34], Figure 5F) were significantly correlated with poor PFS of patients with ovarian cancer. While for the downregulated group, only low expression level of MAOB (*P*=0.0054, HR=0.83 [0.73-0.95], Figure 5I) was associated with poor PFS in patients with ovarian cancer. However, the expression of CDC20, SDC1 and ALDH1A1 were not significantly correlated with PFS of ovarian cancer patients (all *P*>0.05) (Supplementary Figure S2A-C). Therefore, the 5 key genes (UBE2C, TK1, TACC3, CXCR4 and MAOB) were considered for further analysis.

### Detection and validation of upstream miRNAs of the 5 key genes in ovarian cancer

**Figure 6:**
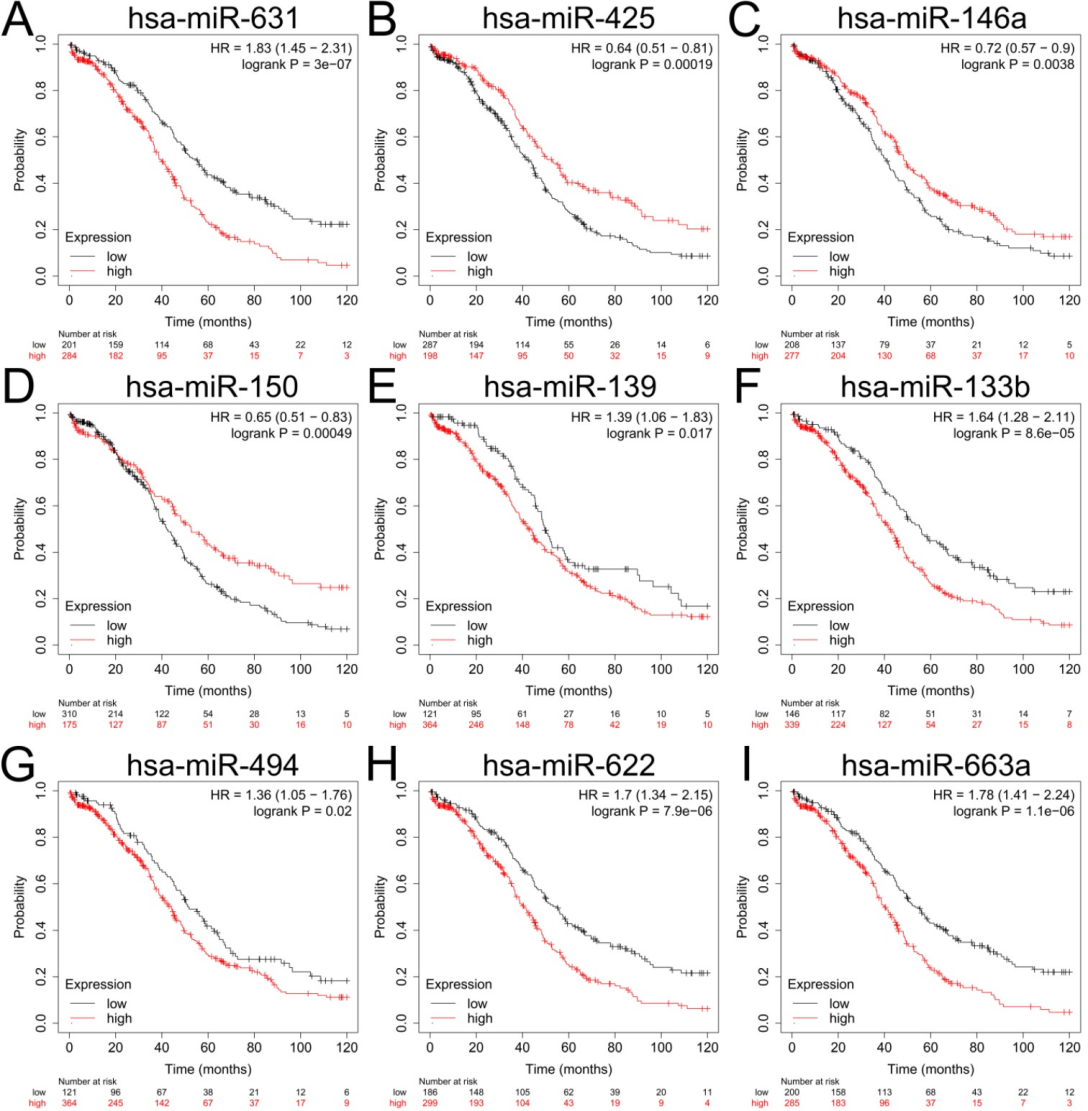
** The prognostic values of upstream downregulated miRNAs in patients with ovarian cancer.** (A-I) Relationship between hsa-miR-631 (A), hsa-miR-425 (B), hsa-miR-146a (C), hsa-miR-150 (D), hsa-miR-139 (E), hsa-miR-133b (F), hsa-miR-494 (G), hsa-miR-622(H), hsa-miR-663a (I) and OS of patients with ovarian cancer by Kaplan-Meier plotter. OS: overall survival.

To explore the upstream miRNAs of those 5 hub genes, miRTarBase were employed to predict the targeted miRNAs of the candidate genes. Based on the filter criteria: only miRNAs verified by at least one powerful experiment were identified as the potential miRNAs interactions (reporter assay, Western blot or quantitative reverse transcription PCR), and a total of 20 upstream miRNAs were eventually identified to be correlated with 3 upregulated hub genes (UBEC2, TACC3 and CXCR4) according to the powerful evidence (Table 3), the upstream miRNAs of TK1 and MAOB were not detected with miRTarBase. Based on the ceRNA hypothesis, the expression of upstream miRNA should be negatively correlated with its target gene, we further evaluated the expression levels and the prognostic values of upstream miRNAs with dbDEMC2 and Kaplan-Meier plotter. The result showed that only downregulation of hsa-miR-425-5p (the upstream miRNA of TACC3) (*P*=0.00019, HR=0.64 [0.51-0.81]), hsa-miR-146a-5p (*P*=0.0038, HR=0.72 [0.57-0.9]) and hsa-miR-150-5p (*P*=0.00049, HR=0.65 [0.51-0.83]) (the upstream miRNA of CXCR4) were correlated with poor overall survival (OS) of patients with ovarian cancer (Figure 6B-D, Supplementary Table S8). The prognosis analysis of other miRNAs was shown in Figure 6, and the three miRNAs (hsa-miR-425-5p, hsa-miR-146a-5p and hsa-miR-150-5p) were regarded as key miRNAs for subsequent exploration.

### Identification and validation of key upstream lncRNAs

**Figure 7:**
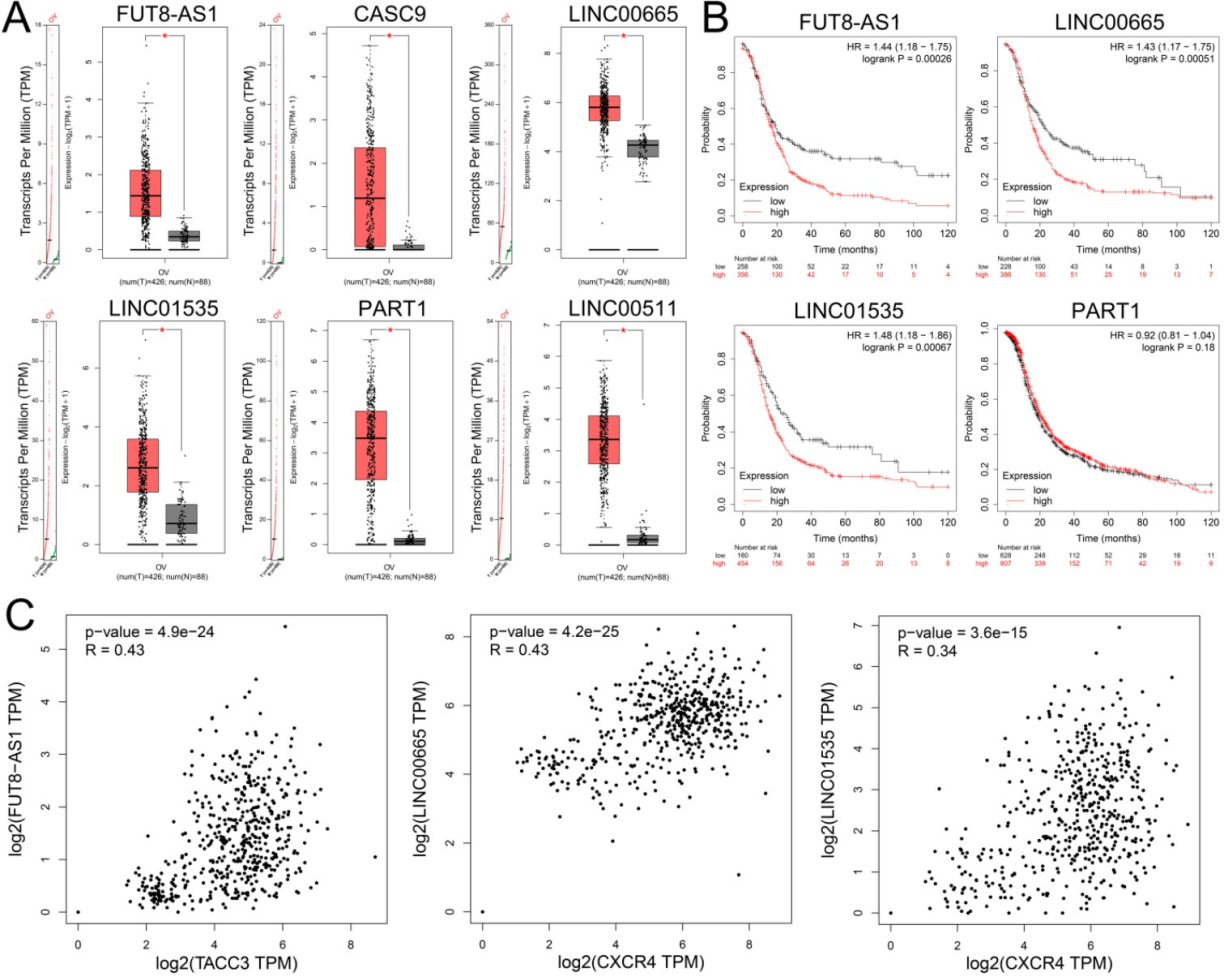
** The expression levels and prognostic values of upstream upregulated lncRNAs in patients with ovarian cancer.** (A) The expression levels of FUT8-AS1, CASC9, LINC00665, LINC01535, PART1 and LINC00511 in ovarian cancer compared with normal samples with GEPIA. (B) The prognostic values of FUT8-AS1, LINC00665, LINC01535, and PART1 in ovarian cancer patients with Kaplan-Meier plotter. (C) The correlation between the key genes (TACC3 and CXCR4) and upstream lncRNAs with GEPIA.

To identify upstream lncRNAs potential binding to the three key miRNAs (hsa-miR-425-5p, hsa-miR-146a-5p and hsa-miR-150-5p), an online database miRNet was used to predict upstream lncRNA. The research showed that a total of 139 lncRNAs were detected in the database for three downregulated miRNAs (Supplementary Table S9). Based on the ceRNA hypothesis, lncRNAs should negatively regulate miRNAs and positively regulated mRNAs. Furthermore, the expression levels of these upstream lncRNAs were detected by GEPIA, and the study demonstrated that only FUT8-AS1, CASC9, LINC00665, LINC01535, PART1 and LINC00511 were upregulated in ovarian cancer compared with normal samples (Figure 7A). Survival analysis showed that high expression of FUT8-AS1, LINC00665 and LINC01535 were significantly correlated with poor OS of patients with ovarian cancer (Figure 7B), CASC9 and LINC00511 were not detected in the Kaplan-Meier plotter database. We further investigated the correlation between lncRNAs and their binding gens with GEPIA, showing that FUT8-AS1 was positively correlated with TACC3 (*P*=4.9e-24, R=0.43), both LINC00665 (*P*=4.2e-25, R=0.43) and LINC01535 (*P*=3.6e-15, R=0.34) were positively correlated with CXCR4 (Figure 7C). As a consequence, FUT8-AS1, LINC00665 and LINC01535 were identified as key upstream lncRNAs of the ceRNA network in ovarian cancer.

### Construction of the lncRNA-miRNA-mRNA regulatory networks in ovarian cancer

**Figure 8:**
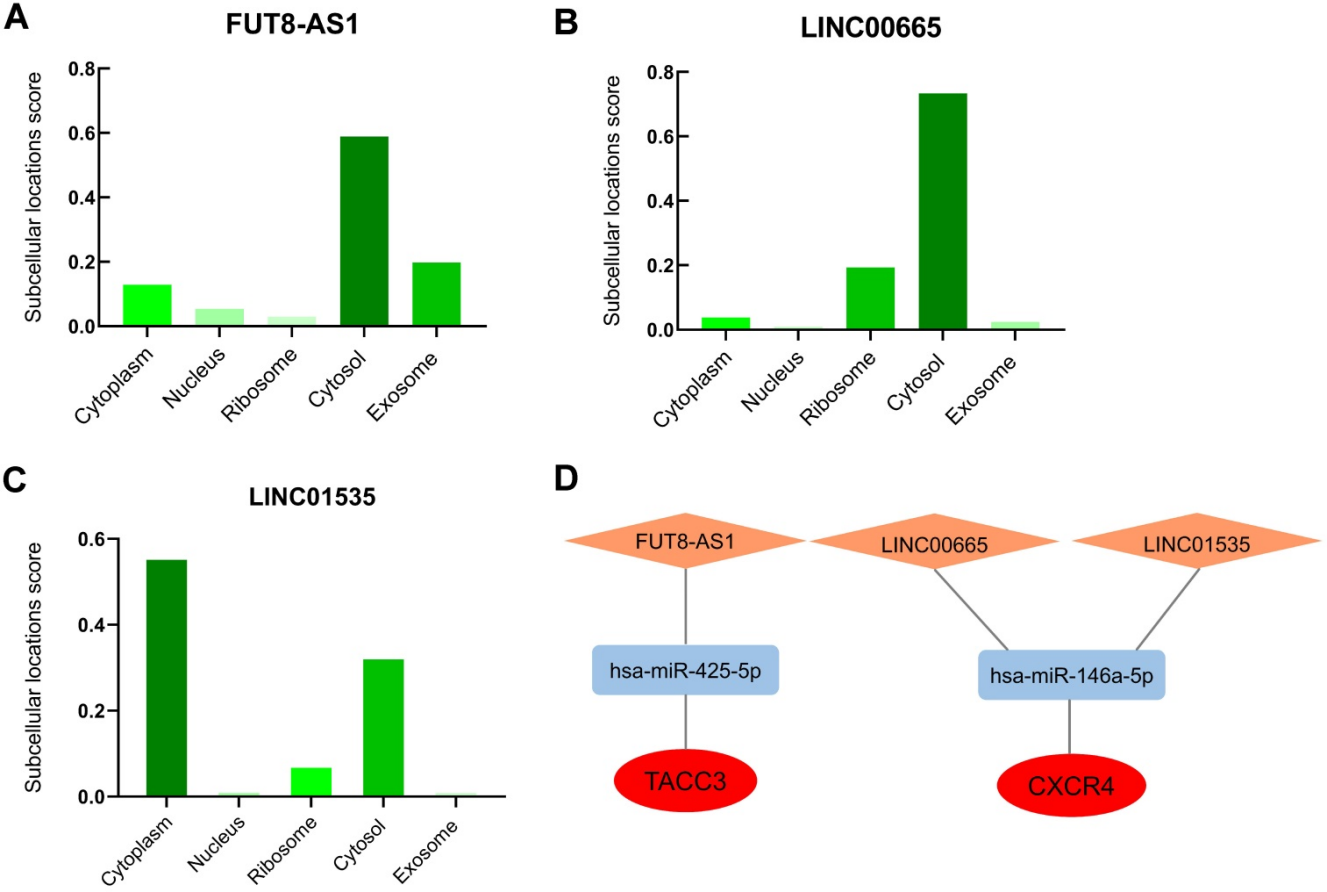
** The potential mRNA-miRNA-lncRNA regulatory network correlated with the prognosis of ovarian cancer.** (A-C) Subcellular localizations of FUT8-AS1 (A), LINC00665 (B) LINC01535 (C) determined by lncLocator. (D) mRNA-miRNA-lncRNA regulatory network correlated with the prognosis of ovarian cancer. TACC3 and CXCR4 in the red ellipse represent upregulated hub genes; has-miR-425-5p and has-miR-146a-5p in the blue rectangle represent downregulated identified miRNAs; FUT8-AS1, LINC00665 and LINC01535 in the orange diamond represent upregulated identified lncRNAs.

The cellular localization of lncRNA exerts profound influence on their molecular functions and mechanisms, we further inverstigated the subcellular localizations of FUT8-AS1, LINC00665 and LINC01535 with lncLocator. The results displayed that FUT8-AS1 and LINC00665 were mainly located in cytosol (score: 0.59 and 0.73, respectively) (Figure 8A-B), LINC01535 was mainly located in cytoplasm and cytosol (score: 0.56 and 0.33, respectively) (Figure 8C), which provided trustworthy evidence that above three lncRNAs may contribute to biological functions and mechanisms through the ceRNA network. According to all the prediction and validation, as shown in Figure 8D, two mRNA-miRNA-lncRNA regulatory networks (TACC3-hsa-miR-425-5p-FUT8-AS1 and CXCR4-hsa-miR-146a-5p- LINC00665/LINC01535) including 2 mRNA (TACC3 and CXCR4), 2 miRNAs (hsa-miR-425-5p, hsa-miR-146a-5p) and 3 lncRNAs (FUT8-AS1, LINC00665 and LINC01535) were eventually constructed and visualized by the Cytoscape software, and each component in the ceRNA network was significantly correlated with the poor prognosis of patient s with ovarian cancer.

We further verified the expression of TACC3 and CXCR4 protein in ovarian cancer tissues and normal tissues stained by immunohistochemistry with HPA. The results showed that TACC3 protein was mainly located to the cell membrane and cytoplasm, and the level of TACC3 proteins were significantly higher in ovarian cancer tissues than those in normal tissues (Supplementary Figure S3). The immunohistochemical staining of CACR4 protein was not detected in HPA.

## Discussion

A variety of complex molecular mechanisms are involved in the tumorigenesis and progression of ovarian cancer, such as abnormal regulation of genes transcription and post-transcription, dysregulation of molecular regulatory network and aberrant activation of signal transduction. The characteristic of tumor markers and mutual regulatory molecules, especially the ceRNA regulatory network 31, are of great significance for evaluating the prognosis of ovarian cancer. Therefore, it is meaningful to screen effective tumor biomarkers and their potential regulatory mechanisms for early diagnosis and prognosis prediction of ovarian cancer.

In the present study, based on GEO database and bioinformatics online tools, we first identified a total of 178 DEGs (38 upregulated and 140 downregulated DGEs) in ovarian cancer and normal samples that were shared by four expression profiles datasets (GSE5438, GSE40595, GSE38666 and GSE26712). Then GO and KEGG enrichment analysis of 178 intersected DEGs were explored with Metascape, the GO enrichment analysis showed that these DEGs were mainly located in extracellular matrix and plasma membrane, and involved in biological functions such as regulating enzyme inhibitor activity, glycosaminoglycan and G protein-coupled receptor binding. KEGG pathway enrichment analysis suggested that DEGs were significantly enriched in signaling pathways such as energy metabolism, proteoglycans in cancer. The above biological behavior and signaling pathways played crucial role in the progression and prognosis of ovarian cancer 32. Therefore, we speculated that these commonly DEGs can affect the occurrence and biological behavior of ovarian cancer through above signaling pathways.

In order to identify the hub genes, two separate PPI networks were investigated with STRING database, and the hub genes were filtered out based on the connectivity degree calculated by Cytoscape software. The top ten upregulated DEGs and top ten downregulated DEGs were further employed for expression validation and survival evaluation. Only high expression of UBE2C, TK1, TACC3 and CXCR4 and low expression of MAOB were proved to be associated with poor prognosis of patients with ovarian cancer, suggesting the five genes were considered to be the key genes in ovarian cancer. Numerous studies have shown that UBE2C overexpression was correlated with poor prognosis and regulated the malignant biological process of various tumors, including endometrial cancer, breast cancer and ovarian cancer 33, 34, 35. It's reported that TACC3 could play an oncogenic role in bladder cancers and prostate cancer 36, 37. Researcher found that CXCR4 was upregulated in colorectal cancer and breast cancer 38, 39, overexpressed CXCR4 promoted the proliferation and invasion of ovarian cancer 40. The studies suggested that these genes were widely involved in occurrence and development of various tumors including ovarian cancer.

We further explored the upstream miRNAs associated with the five hub genes by the online database-miRTarBase based on the potential ceRNA hypothesis. After taking the expression levels and survival exploration into consideration, only three upstream miRNAs were considered as key miRNAs (hsa-miR-425-5p, hsa-miR-146a-5p and hsa-miR-150-5p), all of which were downregulated and associated with poor prognosis in ovarian cancer. Many studies have discovered that these miRNAs functioned as oncogene or tumor suppressor in the tumorigenesis and progression of different tumors. Researchers showed that miR-425-5p inhibited the expression of MALAT1 and TUG1 through inactivating the Wnt/β-catenin signaling pathway and further suppressed the progression of osteosarcoma 41. Iacona* et al*. demonstrated that miR-146a-5p could function as a tumor-suppressive miRNA in lung cancer through targeting EGFR and regulating various metabolic and signaling pathways 42. In addition, miR-146a-5p inhibited the process of EMT by targeting Notch2 in esophageal squamous cell carcinoma 43. It has been reported that miR-150-5p exerted its tumor suppressive functions in breast cancer and colorectal cancer 44, 45, and miR-150-5p was found to be upregulated in ovarian cancer 46. However, there were few researches focused on the function of TACC3-miR-425-5p and CXCR4-miR-146a-5p/ hsa-miR-150-5p in ovarian cancer. Therefore, it is valuable and helpful to explore the potential functions and molecular mechanisms of the miRNAs in ovarian cancer.

Numerous studies have shown that lncRNAs could function as miRNAs sponge to regulate downstream genes based on the ceRNA mechanism, with the upstream lncRNAs binding to the miRNAs further identified with miRNet. After comprehensive evaluation of the expression levels, prognosis values and cellular locations, only high expression of three lncRNAs (FUT8-AS1, LINC00665 and LINC01535) were significantly associated with poor prognosis of ovarian cancer, which were finally proved to be key upstream lncRNAs of the ceRNA network, suggesting that these lncRNAs play crucial in the occurrence and progression of ovarian cancer. Studies focused on FUT8-AS1 were extremely limited. Some studies showed that LINC00665 contributed to the progression and biological behaviors via regulating downstream miRNAs and targeted genes in many malignancies, such as lung cancer, hepatocellular carcinoma, breast cancer 47, 48, 49. Previous research indicated that LINC01535 promoted the proliferation and inhibited the apoptosis of esophageal squamous cell cancer by regulating the JAK/STAT3 signaling pathway 50. Furthermore, LINC01535 contributed to progression of cervical cancer via regulating the miR-214/EZH2 regulatory loop 51. The studies above demonstrated that dysregulations of FUT8-AS1, LINC00665 and LINC01535 were closely associated with the initiation and progression of several tumors.

In consequence, on the basis of ceRNA mechanism, we successfully constructed novel mRNA-miRNA-lncRNA regulatory networks (TACC3-hsa-miR-425-5p-FUT8-AS1 and CXCR4-hsa-miR-146a-5p-LINC00665/LINC01535) associated with prognosis of ovarian cancer. Although existing research may not be perfect, it is valuable to make a conclusion that the ceRNA networks observed in our study could exert profound influences on the predictive accuracy for ovarian cancer, and additional experimental exploration *in vivo* and vitro remains to be carried out to detect the functional mechanisms of ceRNA networks in the future.

## Conclusions

In summary, with a series of integrated bioinformatics databases, we systematically explored and identified DEGs, miRNAs and lncRNAs associated with the prognosis of ovarian cancer. Based on the ceRNA hypothesis, novel mRNA-miRNA-lncRNA regulatory networks (TACC3-hsa-miR-425-5p-FUT8-AS1 and CXCR4-hsa-miR-146a-5p-LINC00665/LINC01535) in ovarian cancer were successfully constructed. The ceRNA networks observed in our study may provide new insights into exploring potential biomarkers for early diagnosis and targeted therapy of ovarian cancer, and further experimental exploration remains to be carried out in the future.

## Supplementary Material

Supplementary figures and tables.

## Figures and Tables

**Table 1:** Details of the four datasets from GEO

Dataset	Platform	Epithelial ovarian cancer	Normal	Reference
GSE54388	GPL570	16	6	Yeung TL et al. (2017)
GSE40595	GPL570	32	6	Yeung TL et al. (2015)
GSE38666	GPL570	18	12	Lili LN et al. (2013)
GSE26712	GPL96	185	10	Bonome T et al. (2018)

**Table 2:** Top 20 upregulated and downregulated DEGs in network ranked by connectivity degree with Cytoscape software

Upregulated DEGs			Downregulated DEGs		
Rank	Name	Score	Rank	Name	Score
1	UBE2C	32	1	KDR	11
1	CDC20	32	2	HSD17B6	7
3	BIRC5	30	3	NANOG	6
4	RNASEH2A	26	3	AOX1	6
5	TK1	24	3	CYP3A5	6
6	TACC3	6	3	ALDH1A1	6
7	CXCR4	2	7	ADH1B	5
7	SDC1	2	7	MAOB	5
7	RNASEH2B	2	7	ALDH1A2	5
7	RNASEH2C	2	10	FGF13	4
7	ANAPC11	2	10	ANXA5	4
12	FXYD3	1	10	HSD17B2	4
12	S100A14	1	10	NR3C2	4
12	KLK8	1	10	ANXA1	4
12	DEFB1	1	10	ADRA2A	4
12	SST	1	16	SCG5	3
12	LCN2	1	16	S100A10	3
12	PTX3	1	16	CPE	3
12	APOA1	1	16	RTN1	3
12	SCGB2A1	1	16	GFPT2	3

**Table 3:** Identification of upstream miRNA of the 5 hub genes in ovarian cancer with miRTarBase

mRNA	miRNA
UBE2C	hsa-miR-20a-5p
	hsa-miR-17-5p
	hsa-miR-631
TK1	-
TACC3	hsa-miR-24-3p
	hsa-miR-152-3p
	hsa-miR-425-5p
CXCR4	hsa-miR-146a-5p
	hsa-miR-146a-3p
	hsa-miR-224-5p
	hsa-miR-150-5p
	hsa-miR-139-5p
	hsa-miR-126-3p
	hsa-miR-9-5p
	hsa-miR-133b
	hsa-miR-494-3p
	hsa-miR-494-5p
	hsa-miR-622
	hsa-miR-204-5p
	hsa-miR-663a
	hsa-miR-335-5p
MAOB	-

## Abbreviations

OC: ovarian cancer
lncRNAs: long noncoding RNAs
ncRNA: noncoding ribonucleic acids
ceRNA: competing endogenous RNA
circRNA: circular RNA
DEG: differentially expressed gene
TCGA: The Cancer Genome Atlas
GEO: Gene Expression Omnibus
PFS: progression free survival
PPI: protein-protein interaction
GO: Gene Ontology
KEGG: Kyoto Encyclopedia of Genes and Genomes
STRING: the Search Tool for the Retrieval of Interacting Genes
TPM: Transcripts per Million
GEPIA: The Gene Expression Profiling Interactive Analysis
KM: The Kaplan-Meier
HPA: The Human Protein Atlas
