# Supplementary material for: Construction of novel mRNA-miRNA-lncRNA regulatory networks associated with prognosis of ovarian cancer
Source: J Cancer. 2020 Oct 17;11(23):7057–72. doi: 10.7150/jca.49557 (PMC7592000; doi:10.7150/jca.49557)
Supplement: Supplementary file 1 — Supplementary figures and tables. [file jcav11p7057s1.pdf]

Table S1. Clinical characteristics of patients with ovarian cancer in GSE38666

Variable	No. (n=18)
Histology	
Serous	18
Stage of disease	
I	2
II	2
III	10
IV	4
Tumor grade	
2	5
3	13

Table S2. Clinical characteristics of patients with ovarian cancer in GSE26712

Variable	No. (n=185)	% or STD[#]
Age at diagnosis	62 yrs.	±12 yrs.
Histology		
Serous	166	90
Adenocarcinoma, NOS	14	8
Mixed	5	3
Stage of disease		
IIIB	8	4
IIIC	136	74
IV	41	22
Tumor grade*		
2	40	22
3	144	78
Surgical outcome		
Optimal	92	50
Suboptimal	93	50

[#]Totals may not equal 100 due to rounding

NOS: not otherwise specified

*Numbers do not sum to the total due to unavailable data

Table S3. Intersected DEGs of four gene expression profiles in ovarian cancer from the Gene Expression Omnibus (GEO)

Upregulated DEGs	CXCR4, BIRC5, KLK8, PRSS21, VTCN1, TMPRSS4, SST, SCGB1D2, UCP2, ADGRG1, TACC3, GLDC, H1-2, BIK, DEFB1, MEOX1, H2BC5, MAL, FAM171A1, S100A14, ASRGL1, PTX3, SCGB2A1, CHODL, TK1, HOMER2, UBE2C, SDC1, PAX8, RNASEH2A, ST6GALNAC2, HEY2, FAM107A, LCN2, APOA1, PRAME, SLC52A2, FXYD3
Downregulated DEGs	KCNJ2, HSD17B2, RTN1, AQP9, FRY, STK26, ANXA3, GFPT2, GHR, SERPINB9, NEFH, N4BP2L2, BCHE, ADRA2A, RGS4, EFHC1, BAMBI, BDH2, DPYD, HLF, TPBG, NDNF, IFI16, ATP7B, RAB38, CYP3A5, DIXDC1, CPVL, LXN, GSDME, SNCAIP, HSPA2, IL18, CALB2, TPD52L1, SLC31A2, MCTP2, NELL2, GPM6A, CCDC28A, REEP1, BNC1, PNISR, LGALS2, NR3C2, CHGB, TACC1, CSGALNACT1, UFSP2, PHACTR2, CBX7, STAR, FCGR3B, PDZD2, CELF2, HBB, PCDH17, PEG3, SLC35G2, FLRT3, HAND2-AS1, ADAMTS3, ALDH1A3, FGF13, PLPP1, SLC46A3, DMD, MARCO, CPE, HPSE, CTSO, PITPNC1, SLIT2, AKAP11, BNC2, SLC4A4, AOX1, AKT3, PROCR, HSD17B6, SLC16A1, RNASE4, KAT2B, TRIM68, CHRDL1, ANXA1, NANOG, TSPAN8, GSAP, ALDH1A2, MNDA, THBD, RSRP1, VLDLR, HBD, CRIM1, SGCG, WASF3, MTUS1, TXNIP, ANXA5, CMAHP, PDPN, DIRAS3, KDR, CLDN15, DAB2, MAOB, CFI, FGF9, C21orf62, ALDH1A1, WNT2B, BICC1, BEX1, ARHGAP44, S100A10, GPRASP1, MPDZ, PCDH9, DIPK1A, ADH1B, ARHGAP6, RADX, UGP2, CXCL6, SEMA5A, LGALS8, HAS1, LHX2, GATM, ARHGEF10, SCG5, DKFZP586I1420, GCNT1, SFRP1, DAPK1, ATF7IP2, SPOCK1

Table S4. Significantly enriched GO annotations (Molecular Functions) of intersected DEGs in ovarian cancer with Metascape

Term	Category	Description	Count	%	Log*P*	Log(q-value
GO:0004857	GO Molecular Functions	enzyme inhibitor activity	14	3.68	-6.05	-2.53
GO:0005539	GO Molecular Functions	glycosaminoglycan binding	11	4.72	-5.90	-2.53
GO:0001758	GO Molecular Functions	retinal dehydrogenase activity	3	37.50	-4.68	-1.71
GO:0001664	GO Molecular Functions	G protein-coupled receptor binding	10	3.48	-4.27	-1.38
GO:0005509	GO Molecular Functions	calcium ion binding	15	2.13	-3.64	-0.89
GO:0055102	GO Molecular Functions	lipase inhibitor activity	3	16.67	-3.54	-0.87
GO:0038024	GO Molecular Functions	cargo receptor activity	5	6.17	-3.49	-0.86
GO:0016491	GO Molecular Functions	oxidoreductase activity	15	2.00	-3.37	-0.78
GO:0019825	GO Molecular Functions	oxygen binding	3	8.33	-2.64	-0.25
GO:0043394	GO Molecular Functions	proteoglycan binding	3	8.33	-2.64	-0.25
GO:0005109	GO Molecular Functions	frizzled binding	3	7.89	-2.57	-0.20
GO:0001540	GO Molecular Functions	amyloid-beta binding	4	4.94	-2.52	-0.18
GO:0042803	GO Molecular Functions	protein homodimerization activity	11	1.70	-2.08	0.00
GO:0016616	GO Molecular Functions	oxidoreductase activity, acting on the CH-OH group of donors, NAD or NADP as acceptor	4	3.25	-1.89	0.00
GO:0015631	GO Molecular Functions	tubulin binding	7	2.02	-1.84	0.00
GO:0008514	GO Molecular Functions	organic anion transmembrane transporter activity	5	2.35	-1.69	0.00
GO:0048018	GO Molecular Functions	receptor ligand activity	8	1.67	-1.59	0.00
GO:0005496	GO Molecular Functions	steroid binding	3	3.16	-1.48	0.00
GO:0030674	GO Molecular Functions	protein binding, bridging	4	2.33	-1.42	0.00
GO:0051087	GO Molecular Functions	chaperone binding	3	2.94	-1.41	0.00

Table S5. Significantly enriched GO annotations (Biological Processes) of intersected DEGs in ovarian cancer with Metascape

Term	Category	Description	Count	%	Log*P*	Log(q-value)
GO:0048545	GO Biological Processes	response to steroid hormone	16	4.15	-7.55	-3.65
GO:0042445	GO Biological Processes	hormone metabolic process	13	5.39	-7.55	-3.65
GO:0030900	GO Biological Processes	forebrain development	14	3.60	-5.93	-2.39
GO:0032970	GO Biological Processes	regulation of actin filament-based process	14	3.47	-5.74	-2.39
GO:0090303	GO Biological Processes	positive regulation of wound healing	6	8.70	-4.93	-1.98
GO:0022604	GO Biological Processes	regulation of cell morphogenesis	14	2.78	-4.66	-1.84
GO:0008406	GO Biological Processes	gonad development	9	4.13	-4.46	-1.69
GO:0042737	GO Biological Processes	drug catabolic process	7	4.83	-4.00	-1.41
GO:0042542	GO Biological Processes	response to hydrogen peroxide	7	4.79	-3.98	-1.41
GO:0061042	GO Biological Processes	vascular wound healing	3	23.08	-3.98	-1.41
GO:0040017	GO Biological Processes	positive regulation of locomotion	14	2.29	-3.75	-1.26
GO:0060485	GO Biological Processes	mesenchyme development	9	3.24	-3.66	-1.21
GO:0031667	GO Biological Processes	response to nutrient levels	12	2.40	-3.47	-1.08
GO:0035584	GO Biological Processes	calcium-mediated signaling using intracellular calcium source	3	13.64	-3.27	-0.96
GO:0106106	GO Biological Processes	cold-induced thermogenesis	6	4.17	-3.16	-0.90
GO:0021871	GO Biological Processes	forebrain regionalization	3	12.50	-3.16	-0.90
GO:0051346	GO Biological Processes	negative regulation of hydrolase activity	11	2.34	-3.14	-0.89
GO:0007266	GO Biological Processes	Rho protein signal transduction	6	4.00	-3.07	-0.87
GO:0006935	GO Biological Processes	chemotaxis	13	2.00	-2.99	-0.86
GO:0060047	GO Biological Processes	heart contraction	8	2.82	-2.91	-0.82

Table S6. Significantly enriched GO annotations (Cellular Component) of intersected DEGs in ovarian cancer with Metascape

Term	Category	Description	Count	%	Log*P*	Log(q-value)
GO:0031012	GO Cellular Components	extracellular matrix	14	2.62	-4.37	-1.08
GO:0072562	GO Cellular Components	blood microparticle	6	4.08	-3.12	-0.32
GO:0016328	GO Cellular Components	lateral plasma membrane	4	6.67	-3.01	-0.32
GO:0016323	GO Cellular Components	basolateral plasma membrane	7	3.10	-2.84	-0.26
GO:0045121	GO Cellular Components	membrane raft	8	2.45	-2.53	-0.08
GO:0031970	GO Cellular Components	organelle envelope lumen	4	4.49	-2.38	-0.04
GO:0098858	GO Cellular Components	actin-based cell projection	6	2.76	-2.27	0.00
GO:0098794	GO Cellular Components	postsynapse	11	1.73	-2.13	0.00
GO:0005911	GO Cellular Components	cell-cell junction	8	2.02	-2.04	0.00
GO:0035580	GO Cellular Components	specific granule lumen	3	4.84	-1.97	0.00
GO:0030055	GO Cellular Components	cell-substrate junction	8	1.92	-1.92	0.00
GO:0005773	GO Cellular Components	vacuole	11	1.38	-1.47	0.00
GO:0043025	GO Cellular Components	neuronal cell body	8	1.57	-1.46	0.00
GO:0005819	GO Cellular Components	spindle	6	1.68	-1.31	0.00

Table S7. Significantly enriched KEGG pathway of intersected DEGs in ovarian cancer with Metascape

Term	Category	Description	Count	%	Log*P*	Log(q-value)
hsa00830	KEGG Pathway	Retinol metabolism	6	9.23	-5.09	-2.39
hsa05143	KEGG Pathway	African trypanosomiasis	3	8.57	-2.67	-0.58
hsa00260	KEGG Pathway	Glycine, serine and threonine metabolism	3	7.50	-2.50	-0.52
hsa05205	KEGG Pathway	Proteoglycans in cancer	6	2.96	-2.41	-0.52
hsa05224	KEGG Pathway	Breast cancer	5	3.47	-2.37	-0.52
hsa00140	KEGG Pathway	Steroid hormone biosynthesis	3	5.17	-2.05	-0.36
hsa01524	KEGG Pathway	Platinum drug resistance	3	4.11	-1.78	-0.19
hsa04610	KEGG Pathway	Complement and coagulation cascades	3	3.80	-1.69	-0.17
hsa04666	KEGG Pathway	Fc gamma R-mediated phagocytosis	3	3.30	-1.53	-0.07
hsa05164	KEGG Pathway	Influenza A	4	2.31	-1.41	0.00
hsa04060	KEGG Pathway	Cytokine-cytokine receptor interaction	5	1.85	-1.31	0.00

Table S8. The expression levels of upstream miRNA of UBE2C, TACC3 and CXCR4 in ovarian cancer analyzed by dbDEMC2

miRBase ID	GEO series	Cancer Type	logFC	P value	adj *P* value	Status
hsa-miR-20a-5p	GSE65819	ovarian cancer	2.587222928	3.18997E-05	0.00029	UP
hsa-miR-17-5p	GSE31801	ovarian cancer	-0.396563981	0.000154431	0.00040318	DOWN
hsa-miR-631	GSE31801	ovarian cancer	0.397232845	2.79723E-08	1.7025E-07	UP
hsa-miR-24-3p	GSE65819	ovarian cancer	3.092357611	1.20321E-07	3.8503E-06	UP
hsa-miR-152-3p	GSE31801	ovarian cancer	-0.179306902	1.73132E-06	6.781E-06	DOWN
hsa-miR-425-5p	GSE31801	ovarian cancer	-0.102414916	0.016710923	0.02856049	DOWN
hsa-miR-146a-5p	GSE31801	ovarian cancer	-0.042143125	0.000518804	0.00120579	DOWN
hsa-miR-146a-3p	-	-	-	-	-	-
hsa-miR-224-5p	GSE47841	ovarian cancer	2.282826333	0.000739712	0.00556042	UP
hsa-miR-150-5p	GSE31801	ovarian cancer	-0.616267623	3.91214E-14	9.0306E-13	DOWN
hsa-miR-139-5p	GSE31801	ovarian cancer	-0.298779024	3.21634E-09	2.4737E-08	DOWN
hsa-miR-126-3p	GSE65819	ovarian cancer	2.458895525	0.013959152	0.03891053	UP
hsa-miR-9-5p	GSE31801	ovarian cancer	-0.354997513	0.024396224	0.04007613	DOWN
hsa-miR-133b	GSE31801	ovarian cancer	0.051733679	0.022400713	0.03708611	UP
hsa-miR-494-3p	GSE65819	ovarian cancer	-1.222629947	0.011303539	0.03229582	DOWN
hsa-miR-494-5p	-	-	-	-	-	-
hsa-miR-622	GSE65819	ovarian cancer	-1.687035266	0.018731808	0.04978554	DOWN
hsa-miR-204-5p	GSE47841	ovarian cancer	-1.72920938	3.23762E-07	8.5246E-06	DOWN
hsa-miR-663a	GSE31801	ovarian cancer	0.441219262	3.63184E-15	9.9114E-14	UP
hsa-miR-335-5p	GSE31801	ovarian cancer	0.763349212	7.51162E-18	3.7381E-16	UP

Table S9. Identification of upstream lncRNAs of the 3 key miRNAs in ovarian cancer with miRNet

miRNA	Target LncRNA
hsa-mir-425-5p	LINC01806, LINC00608, THUMPD3-AS1, SNHG8, LINC02219, LINC01184, HCG18, HCG22, TRAF3IP2-AS1, PRKAG2-AS1, MSC-AS1, SNHG7, LINC00858, KCNQ1OT1, NEAT1, MALAT1, KCTD21-AS1, LINC02396, LINC00641, PSMA3-AS1, FUT8-AS1, TCL6, SNHG14, GABPB1-IT1, NR2F2-AS1, SNHG16, LINC01977, LINC00662, LINC00159, MCM3AP-AS1, TTC28-AS1, TUG1, XIST, ARMCX5-GPRASP2, LINC00630, LINC00894
hsa-miR-146a-5p	LINC00115, MIR137HG, MIR181A1HG, MIR29B2CHG, ITPKB-IT1, MIR4453HG, TMEM161B-AS1, EPB41L4A-AS1, LINC00847, ZSCAN16-AS1, HCG18, LINC02538, LINC01176, STAG3L5P-PVRIG2P-PILRB, CASC9, AZIN1-AS1, MINCR, EBLN3P, FAM201A, LINC00963, SNHG7, CCDC183-AS1, OLMALINC, KCNQ1OT1, NEAT1, MALAT1, MIR100HG, LINC00567, SLC25A21-AS1, LINC02288, ZNF710-AS1, ZNF213-AS1, CRNDE, TBX2-AS1, SNHG16, LINC00665, LINC01535, ZNF337-AS1, LINC01311,XIST
hsa-miR-150-5p	LINC01128, PINK1-AS, FOXD2-AS1, IL6R-AS1, RN7SL832P, LINC01121, LINC01798, BOLA3-AS1, PAX8-AS1, EIF1B-AS1, PTPRG-AS1, TMCC1-AS1, CLRN1-AS1, MUC20-OT1, NNT-AS1, PART1, SCAMP1-AS1, TMEM161B-AS1, LMNB1-DT, LINC00847, HCG18, LINC00174, LINC00689, OTUD6B-AS1, ARRDC1-AS1, LINC01553, ENTPD1-AS1, NEAT1, MALAT1, MIR4697HG, PPP1R12A-AS1, LINC02302, LINC01588, PSMA3-AS1, VASH1-AS1, LINC02292, FAM30A, SRP14-AS1, OIP5-AS1, GABPB1-IT1, GABPB1-AS1, LINC02568, USP3-AS1, NPTN-IT1, CPEB1-AS1, MMP25-AS1, MIR762HG, LHX1-DT, LINC00511, NORAD, ZFAS1, DSCAM-AS1, TSPEAR-AS2, DGCR12, LINC00895, MIAT, OGFRP1, MIRLET7BHG, C22orf34, XIST, FTX, LINC01285, TTTY15

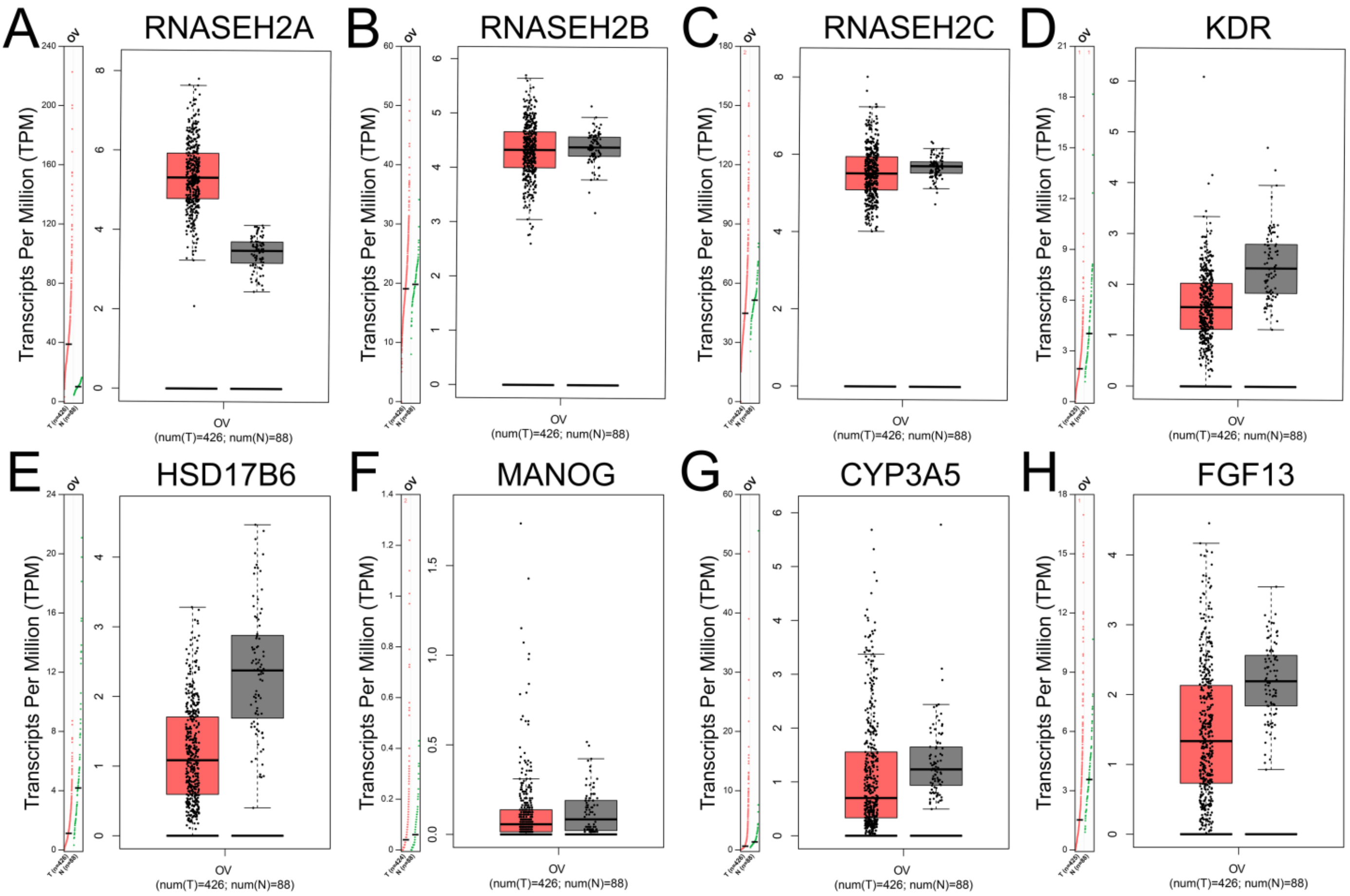

Figure S1. Expression levels of hub genes in patients with ovarian cancer validated with GEPIA. (A-H) The expression levels of hub genes RNASEH2A (A), RNASEH2B (B), RNASEH2C (C), KDR (D), HSD17B6 (E), MANOG (F), CYP3A5 (G) and FGF13 (H) in ovarian cancer compared with normal tissues (all P<0.05).

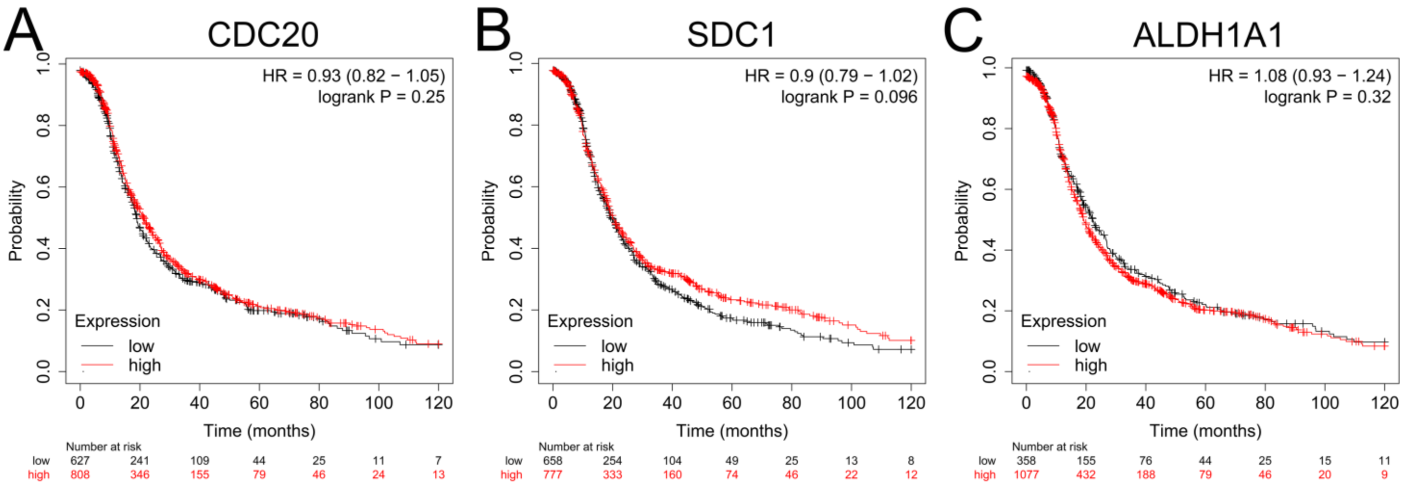

Figure S2. Prognostic values of hub genes in ovarian cancer analyzed by using the Kaplan–Meier plotter. (A-C) Relationship between CDC20 (A), SDC1 (B), ALDH1A1 (C) and PFS of patients with ovarian cancer.

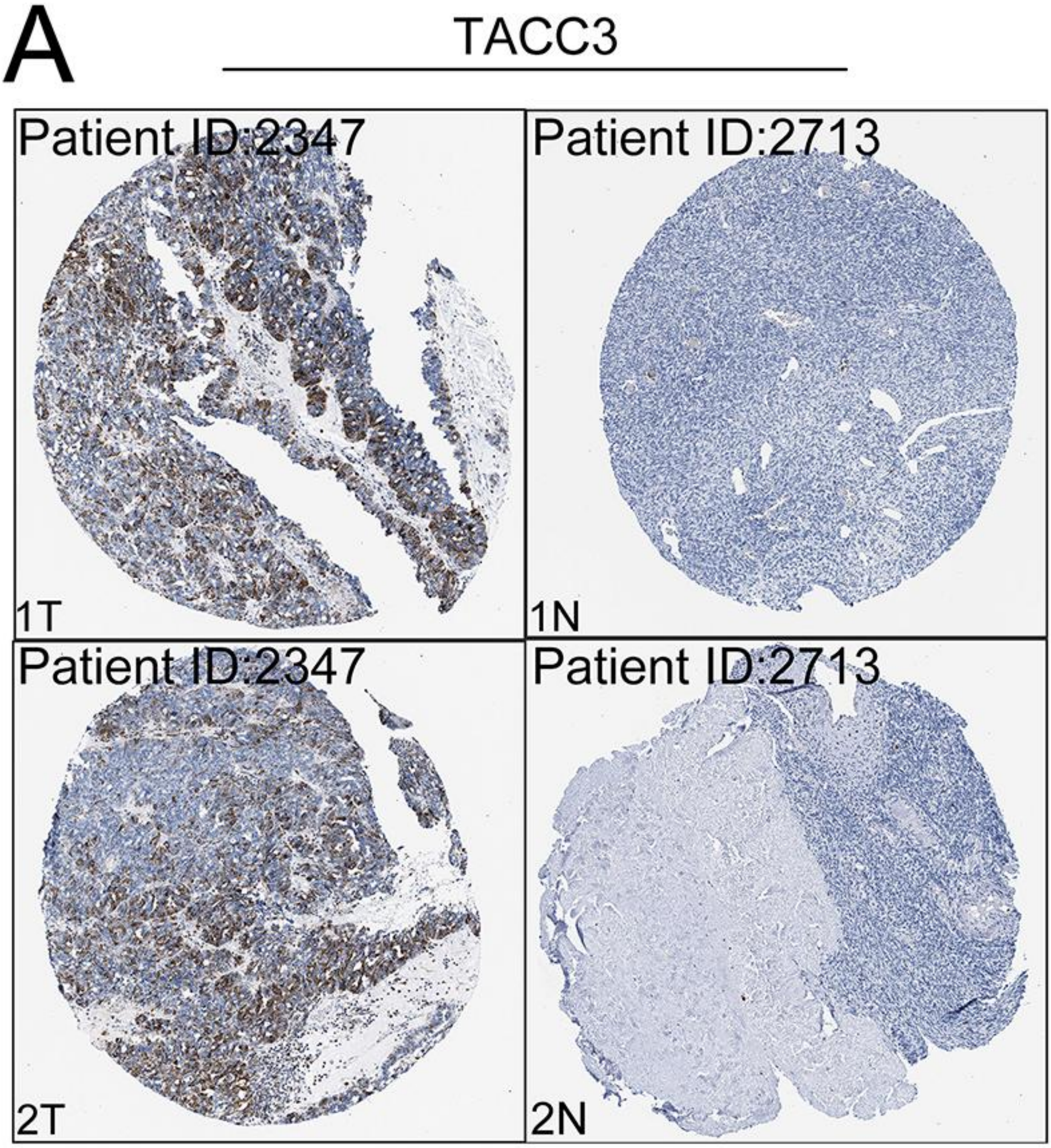

Figure S3. The expression of TACC3 protein in ovarian cancer (Human Protein Atlas). (A) The expression of TACC3 in ovarian cancer (staining: High; intensity: Strong; quantity: 75–25%; Location: Cytoplasmic/membranous) and normal tissues (staining: Not detected; intensity: Negative; quantity: None; Location: None) stained by immunohistochemistry. T: Tumor; N: Normal.
